# Barriers and Facilitators of Parental Involvement in Applied Behavior Analysis: A Phenomenological Study

**DOI:** 10.3390/bs16071134

**Published:** 2026-07-06

**Authors:** Andres F. Mambuca

**Affiliations:** Mental Health Institute of Florida, Pembroke Pines, FL 33028, USA; clinicaldirector@mhiof.com

**Keywords:** autism, applied behavior analysis, parental involvement, caregiver engagement, family systems theory, qualitative research, reflexive thematic analysis, service design, underserved populations

## Abstract

Parental involvement in applied behavior analysis (ABA) interventions for children with autism spectrum disorder (ASD) is essential for treatment success, yet caregiver engagement remains inconsistent across clinical contexts. This qualitative study employs reflexive thematic analysis to explore the lived experiences of eight mothers receiving ABA services through a single South Florida provider. We identified barriers (time constraints, insufficient family support, communication gaps) and facilitators (observed child progress, economic support, intrinsic motivation) to parental involvement. Interpreted through a family systems lens, findings suggest that parental engagement is better understood as a dynamic, context-dependent process shaped by systemic and relational factors rather than as a matter of individual motivation alone. This modest, single-site account contributes contextually to understanding how service-design factors shape caregiver participation within one ABA program and aligns with existing literature on barriers to service engagement among underserved families. Clinical implications point to concrete service adaptations including flexible scheduling, enhanced communication protocols, family-centered psychoeducation, and evaluation of caregiver-recommended practices such as relaxation techniques and family therapy. While this study cannot generalize broadly, its findings offer specific recommendations for improving practice in community-based ABA settings serving diverse populations.

## 1. Introduction

Autism spectrum disorder (ASD) is a neurodevelopmental condition characterized by persistent deficits in social communication and the presence of restricted and repetitive patterns of behavior (American Psychiatric Association, 2013). Over recent decades, the prevalence of ASD has increased substantially, intensifying demand for effective, evidence-based interventions and placing considerable strain on families and healthcare systems.

Applied behavior analysis (ABA) has emerged as one of the most empirically supported interventions for individuals with ASD. ABA applies behavioral principles systematically to promote adaptive functioning and reduce maladaptive behaviors. However, the effectiveness of ABA is not determined solely by clinical procedures; it is significantly influenced by contextual variables, particularly caregiver involvement. Research has consistently demonstrated that parental involvement is associated with enhanced skill generalization, increased treatment fidelity, and greater long-term maintenance of behavioral gains (Bearss et al., 2015; Kazdin, 2005; McConachie & Diggle, 2007).

Despite the documented importance of parental involvement, engagement remains inconsistent across clinical contexts. Caregivers frequently encounter barriers such as time constraints, occupational demands, emotional stress, and limited support systems, while systemic factors such as communication difficulties between providers and families further complicate engagement. Understanding these barriers is important for clinical practice; however, much of the existing literature examines engagement through the lens of individual caregiver characteristics (motivation, stress, competence) rather than through the lived experiences and perspectives of caregivers themselves. This study seeks to address that gap by centering caregiver voice and exploring how parents themselves describe and prioritize the factors that shape their involvement in ABA services.

Family systems theory offers a useful framework for understanding these dynamics. This perspective, particularly as articulated by Bowen (1978), conceptualizes the family as an interconnected system in which individual behaviors are shaped by relational and environmental influences. Within this framework, parental involvement is not solely an individual responsibility but a function of broader systemic interactions—spousal participation, extended-family beliefs about therapy, the distribution of caregiving load, and structural supports such as access to childcare and financial resources. This systems-level analysis is essential for designing interventions that respond to the actual constraints families face, rather than attributing disengagement to individual deficits of motivation or competence.

The present study explores the lived experiences of eight mothers involved in ABA interventions within a single South Florida ABA program. This is a modest, qualitative, single-site account that contributes contextually to understanding how systemic and relational factors shape caregiver participation. We do not claim to offer generalizable conclusions about parental involvement in ABA more broadly, nor do we suggest that these findings apply uniformly to other ABA programs, geographic regions, or populations. Rather, we provide a detailed exploration of how one group of mothers, many from Hispanic and socioeconomically diverse backgrounds, describe their experiences and recommend improvements to service delivery. This contextually grounded approach is appropriate for a qualitative descriptive study and aligns with the epistemological commitments of reflexive thematic analysis.

The study addresses four research questions:

- (RQ1) What barriers prevent parental involvement in treatment?
- (RQ2) What factors promote involvement?
- (RQ3) How does participation contribute to treatment success?
- (RQ4) What changes would caregivers recommend to ABA services?

## 2. Materials and Methods

### 2.1. Research Design

This study employed a qualitative descriptive design using reflexive thematic analysis to explore caregivers’ experiences of involvement in ABA services. We explicitly situate this work within the tradition of qualitative description and reflexive thematic analysis (Braun & Clarke, 2006, 2019), not phenomenology, as the title might suggest. Qualitative description is appropriate when the research goal is to understand participants’ lived experiences in relation to a specific phenomenon (parental involvement in ABA) without the requirement for deep phenomenological reduction or the identification of essential structures. Reflexive thematic analysis allows for the systematic identification of patterns (themes) within qualitative data while maintaining awareness of how the researcher’s own perspectives and assumptions shape the analysis. This approach is particularly well suited to research seeking to understand contextual, systemic factors in caregiving and service engagement.

### 2.2. Reflexivity and Researcher Positioning

The primary researcher is a board-certified behavior analyst (BCBA) and licensed mental health counselor (LMHC) with extensive clinical experience in ABA-based treatment for children with autism. Consistent with the epistemological commitments of reflexive thematic analysis (Braun & Clarke, 2019), researcher subjectivity was treated not as a confound to be neutralized but as an active analytical resource. A reflexive journal was maintained throughout data familiarization, initial coding, and theme development. Journal entries documented pre-existing clinical assumptions—including an initial assumption that caregiver non-attendance reflected motivational deficits—and tracked how those assumptions were interrogated, modified, or overturned by engagement with participant accounts.

Two specific moments of interpretive revision are instructive. First, an early interpretive code of ‘caregiver avoidance’ was reconsidered after sustained engagement with P06’s account. Initial reading suggested she was avoiding session attendance; however, closer attention to her descriptions of communication patterns revealed that her reduced participation reflected constrained engagement due to communication asymmetry between the family and the clinical team, not avoidance per se. Second, an initial reading of P04’s account as reflecting individual caregiver burden was revised to foreground subsystem-level conflict after examining the role of the maternal grandmother’s counter-therapeutic influence within the family system. These moments of interpretive revision are central to the analytic process in RTA and are reported here to make the researcher’s role in meaning-making transparent rather than to claim that subjective influence was neutralized.

### 2.3. Ethical Considerations

This study is a secondary analysis of qualitative data originally collected for a doctoral dissertation at California Southern University. Institutional Review Board (IRB) approval was obtained prior to the original data collection (protocol approval date: 13 April 2021). No new data were collected for the present study. Only participants with documented informed consent were included, and all identifying information was removed to ensure confidentiality.

### 2.4. Participants

Participants were eight mothers (P01–P08) of children diagnosed with ASD, all actively receiving ABA services through the Mental Health Institute of Florida at the time of original data collection. Participants were recruited via convenience sampling. Inclusion required direct involvement in the child’s treatment and sufficient experience with ABA to provide meaningful insight. All participants were U.S. citizens or U.S.-born, resided in Broward or Miami-Dade counties, and were fluent in English. Household income ranged widely ($15,000 to $137,000 annually), and the sample was predominantly Hispanic in cultural background, reflecting the demographic profile of the catchment area.

While all participants were U.S. citizens or U.S.-born, resided in Broward or Miami-Dade counties, and were fluent in English, detailed child-level and treatment-level information was not systematically collected in the original protocol. Systematic data on child age, ASD severity or support needs, behavioral profile, duration of ABA services, treatment intensity, and type of parental training received were not available. The absence of these variables represents a significant analytical constraint, which is discussed in the Limitations section (Section 5). Readers should interpret all findings with these constraints in mind. This limitation reflects the reality that the manuscript represents a secondary analysis of data collected with a different primary focus; future research should systematically collect child- and treatment-level variables to enable more nuanced interpretation of how barriers operate across different service contexts and family configurations.

Sample size was evaluated using the concept of information power (Malterud et al., 2016). Given the study’s focused aim, its specific sample, and a theory-informed analysis, eight in-depth interviews provided adequate information power. Importantly, this study includes only mothers; the perspectives of fathers, grandparents, or other caregivers are absent. Additionally, this is a convenience sample from a single service provider, which limits the diversity of service contexts and ABA program models represented.

### 2.5. Data Collection

Data were collected through semi-structured interviews lasting approximately 30 to 60 min, conducted by telephone and audio-recorded for transcription. A nine-item interview guide explored caregiver experiences, perceived barriers, motivating factors, role perceptions, and recommended service changes. The interview guide is reproduced in Appendix A. Important limitation: The original interview protocol, while comprehensive in exploring barriers and facilitators, was not specifically designed to collect systematic information about the children (age, ASD severity, behavioral profile, support needs) or treatment characteristics (intensity, duration, type of parental training). This absence of child- and treatment-level data constrains the analysis, as these variables may strongly influence how time constraints, family support, communication, and economic barriers operate in different families.

### 2.6. Data Analysis

Transcripts were analyzed using reflexive thematic analysis following Braun and Clarke (2006, 2019). The researcher (1) achieved familiarization through repeated reading; (2) coded all statements relevant to the research questions; (3) generated initial codes; (4) clustered codes into candidate themes; (5) reviewed themes against the full dataset; and (6) defined and named final themes. Verbatim quotations were retained to evidence each theme. Codes were not predetermined but emerged from close engagement with the data. The analytical process was iterative: as themes solidified, the researcher returned to the full dataset to ensure that no relevant material had been overlooked and that coded material was accurately represented by the theme it was assigned to.

### 2.7. Trustworthiness

Credibility was supported through member checking. Preliminary theme summaries (not full transcripts, not final interpretations) were returned to all eight participants following initial analysis. Six of the eight participants responded within the follow-up window. Responses were generally confirmatory; no participant disputed the accuracy of their attributed quotations. Two participants provided elaborating comments that informed minor adjustments to the analysis. P04 offered additional context regarding the role of the maternal grandmother that reinforced the subsystem-conflict interpretation in Section 3.1.2. P07 placed greater emphasis on school-provider communication barriers than her original interview had suggested, contributing to a modest expansion of the communication theme (Section 3.1.3). No themes were substantially altered as a result of member checking, suggesting that the preliminary analysis had captured participants’ primary concerns with reasonable accuracy.

Confirmability was addressed through the reflexive journaling process described in Section 2.2, which documents the researcher’s interpretive decisions and the points at which initial codes were revised. This creates an auditable trail of analytic choices. Dependability was supported by consistent use of the same semi-structured interview protocol across all participants, permitting comparison across accounts. Transferability is supported by thick description of the sample, service context, and analytical approach; however, given the single-site, convenience sample design, transferability to other ABA programs or populations should be undertaken with caution and with explicit acknowledgment of contextual differences.

## 3. Results

Thematic analysis produced nine themes across the four research questions. Themes emerged as interconnected dynamics that collectively shaped caregiver participation. Each theme is presented with an interpretive claim, supporting quotations from participants, and an explicit connection to the family systems framework.

### 3.1. Barriers to Involvement (RQ1)

#### 3.1.1. Lack of Time to Participate

Rather than reflecting a simple scheduling problem, limited time emerged as a structurally produced barrier: mothers who worked full-time outside the home found that ABA session schedules were designed around assumptions of caregiver availability that many in this sample could not meet. As P01 explained, ‘Time could be a limiting factor. The therapy sessions are long, and I cannot stay all the time participating.’ Limited time was the most consistently reported barrier, driven by work schedules and the logistics of attending sessions.

P02 faced a compounded constraint, with no spousal backup due to her partner’s military deployment: employment outside the home directly displaced participation. P05 described commuting between worksite and clinic as the primary logistical barrier. Time limitations were not merely logistical; they reduced the opportunity to build procedural competence required for home implementation.

It is important to note that ‘time’ as a barrier did not operate uniformly across participants. For P02, the constraint was structural and gendered: as the sole available caregiver in a household where her partner’s military service precluded involvement, her time burden was compounded by the complete absence of co-parenting support. For P05, the barrier was logistical—the geography of commuting between employment and clinic imposed costs that a closer service location might have mitigated. For P01, the barrier was intrinsic to the service model itself: the length of ABA sessions exceeded what her schedule permitted, regardless of logistical support. These distinctions matter for clinical response: they suggest that flexible scheduling alone would address some but not all forms of time constraint and that interventions must be tailored to the specific mechanism through which time functions as a barrier for each family.

Interpreted through a family systems lens, these accounts do not point to insufficient maternal motivation; they point to structural misalignment between service-delivery models and the employment realities of working-class and single-caregiver families. From a systems perspective, the problem is not the mother but the mismatch between program design assumptions and family structural realities.

#### 3.1.2. Lack of Family Support

The absence or misalignment of family support functioned as a systemic obstacle operating at the subsystem level. P04 described two simultaneous sources of strain: ‘The lack of participation of the family—for example, my son’s father never participates in that therapy, and my son’s maternal grandmother always tries to impose her own ideas about the treatment.’ This statement reveals two distinct subsystem-level dynamics that a family systems analysis can illuminate.

First, the father’s non-participation creates an asymmetric caregiving arrangement in which the mother bears exclusive responsibility for treatment implementation across home settings, increasing her burden and reducing the consistency with which behavioral strategies are applied. Second, the grandmother’s active counter-influence introduces competing behavioral contingencies: the child may experience incompatible response demands depending on which caregiver is present, creating inconsistency that is widely recognized in the behavioral literature as detrimental to skill acquisition and maintenance (Osborne et al., 2008).

Several caregivers experienced this as a systemic obstacle rather than an individual shortfall. Interpreted through a family systems lens, these accounts show involvement constrained not by the mother’s willingness but by misalignment among family subsystems. From this perspective, clinical responses that address only the mother’s skills, without engaging the full family system, are likely to have limited generalization value. Interventions that target the family system—engaging fathers, educating extended family members, establishing behavioral consistency across caregivers—would be more likely to produce sustainable change.

#### 3.1.3. Communication Gaps Among Parents, Providers, and Schools

Communication gaps between families and providers were not experienced by participants as neutral informational deficits; they were often experienced through a lens of institutional power asymmetry. Caregivers reported insufficient communication across systems surrounding the child. P07 called for schools to share responsibility: ‘School officials like teachers should be involved and have more knowledge on how to deal with these kinds of children.’ This recommendation reflects not only a desire for coordination but also an implicit critique of the expert–layperson dynamic in which caregivers are positioned as recipients of professional guidance rather than as collaborative partners.

P06 proposed a dedicated professional to interpret the child’s needs for the whole family. Her suggestion reflects that she experienced the informational architecture of the service system as one in which knowledge flowed from professionals to families, rather than in both directions. These dynamics have implications for caregiver self-efficacy and shared decision-making: when mothers perceive that their observations, expertise, and recommendations carry less weight than those of professional providers, their willingness to assert themselves in treatment planning may diminish, reducing the collaborative quality of decision-making and the likelihood that services will be responsive to family-specific needs.

Addressing communication gaps, therefore, requires more than information provision; it requires relational conditions in which parents experience themselves as legitimate co-authors of the treatment process rather than as passive recipients of expert guidance. Communication gaps also affect treatment fidelity: when caregivers do not understand the rationale for specific strategies, or when they perceive the provider’s expectations as misaligned with their family’s needs, they are less likely to implement procedures consistently at home. These accounts link communication to caregiver confidence, shared decision-making, treatment fidelity, and perceived power imbalance between professionals and families.

### 3.2. Facilitators of Involvement (RQ2)

#### 3.2.1. Motivation from Observed Child Progress

Every participant linked motivation to visible improvement in their child. P01 framed her participation around concrete goals: ‘My main motivation to participate in ABA therapy is to help my son get more social inclusion, better grades at school, and better interaction with his family.’ Across the sample, motivation was abundant; what varied were the structural conditions that allowed it to translate into participation.

P06 described how challenging behaviors increased rather than diminished her drive to engage: observing her child’s progress gave her confidence that ABA was working and renewed her commitment to attendance. Across the sample, participants did not articulate skepticism about ABA or doubt that their involvement mattered. Rather, their primary struggle was translating strong motivation into consistent participation despite structural barriers—time, transportation, and competing demands.

This finding reframes the problem of caregiver engagement. Disengagement is better explained by implementation demands placed on families than by any deficit of caregiver motivation. Motivation alone, without addressing the structural barriers to translating motivation into action, is insufficient to ensure consistent participation.

#### 3.2.2. Economic and Structural Support

Caregivers repeatedly identified economic and structural support as enabling conditions for participation. P08 made the mechanism explicit: ‘More economic help would permit me to work fewer hours and dedicate more time to my child’s therapy.’ P03 connected funding to time directly. P07 called for financial help from the government. These accounts position engagement as conditioned by access to time, transportation, and funding rather than by individual choice alone.

Structural and socioeconomic inequality is integral to this account. The wide household-income range in the sample ($15,000–$137,000), its predominantly Hispanic composition, and recurrent requests for governmental and financial assistance indicate that engagement is conditioned by access to time, transportation, and funding. Caregivers with fewer resources face steeper structural barriers. For some, transportation to the clinic consumed a significant portion of household resources; for others, the need to maintain full-time employment to afford basic necessities left no time for session attendance. These are not individual deficits; they are structural constraints imposed by economic inequality.

#### 3.2.3. Parental Role in Treatment Success (RQ3)

All participants regarded their role as integral to treatment success. P01 observed: ‘My son gets more motivation in therapy when I am participating.’ P06 described herself as the person who must ultimately lead the therapy. P04 noted: ‘The treatment gives me the tools to properly manage my son’s behaviors, so in this way I can help him better when the therapists are gone.’ Caregiver involvement functioned as a mechanism of generalization, equipping parents to maintain gains across settings and time.

Participants understood that consistency across settings—home, school, clinic—was essential for their child to generalize new skills. Their involvement was not conceived as supplementary or optional but as foundational to treatment success. This universal recognition of their essential role distinguishes this sample: disengagement was not rooted in disagreement about the value of their participation but in the practical impossibility of maintaining it given structural constraints.

#### 3.2.4. Recommended Changes to ABA Services (RQ4)

Several caregivers proposed incorporating relaxation routines to prepare children for sessions. P03 suggested ‘relaxation exercises before beginning the therapy’ to calm her child before demanding behavioral work. Caregivers also recommended embedding family-oriented support within ABA. P04 proposed that ‘the addition of family therapy sessions could be very beneficial to the overall progress of the child.’ These caregiver-generated proposals point toward a more systemic, family-centered model of service delivery.

Other recommendations included improved communication with schools, flexible scheduling, and financial assistance. These recommendations, derived from caregiver expertise grounded in lived experience, merit serious consideration and formal evaluation as potential adjuncts to standard ABA service delivery.

## 4. Discussion

This study explored the lived experiences of eight mothers involved in ABA services within a single South Florida program. Three barriers recurred consistently—limited time, insufficient family support, and communication gaps—while motivation rooted in observed child progress, together with economic and structural support, emerged as principal facilitators of involvement. These findings are situated within a family systems theoretical framework that conceptualizes parental involvement as a property of the family system rather than as an individual trait or responsibility.

Every participant expressed strong motivation to participate in their child’s ABA treatment; what constrained them were structural and relational conditions—session length, scheduling demands, unequal caregiving loads, and limited buy-in from partners and extended family. The accounts of P04 and P06, in which a non-participating father and an authority-claiming grandmother complicate engagement, exemplify how subsystem misalignment within the family limits the primary caregiver’s participation. These findings invite a critical reflection on ABA service delivery.

### 4.1. How Barriers Operate Across the Family System

The data suggest that disengagement is better explained by implementation demands placed on families than by any deficit of caregiver motivation or competence. Long daily sessions, travel between work and home, and limited family education impose requirements that many working, single, or lower-income caregivers cannot meet without support. Framing involvement as a service-design problem, rather than a caregiver-deficit problem, reorients the field toward modifiable structural targets (Brookman-Frazee et al., 2006; Karst & Van Hecke, 2012).

Structural and socioeconomic inequality is integral to this account. The wide household-income range in the sample ($15,000–$137,000), its predominantly Hispanic composition, and recurrent requests for governmental and financial assistance indicate that engagement is conditioned by access to time, transportation, and funding. Caregivers with fewer resources face steeper structural barriers. This finding aligns with the literature on service equity and access disparities in early intervention for diverse and underserved populations (Zablotsky et al., 2014).

### 4.2. Clinical Recommendations Derived from Findings

The following clinical recommendations are grounded in specific themes and participant accounts. Each recommendation is provisional, based on a small qualitative sample, and should be tested in larger, more diverse samples before being adopted as evidence-based practice guidelines.

Clinical Recommendation 1 (Flexible Scheduling) is directly motivated by Section 3.1.1 (Lack of Time), in which time constraints driven by employment, commuting, and session length were identified as the most consistently reported barrier. ABA programs should explore options such as shorter, more frequent sessions; end-of-day or weekend scheduling; and telehealth coaching segments that allow parents to receive guidance without requiring full-session attendance. Stahmer et al. (2010) demonstrate that flexible delivery models can maintain fidelity while reducing family burden.

Clinical Recommendation 2 (Family-Inclusive Psychoeducation) is motivated by Section 3.1.2 (Lack of Family Support), particularly the accounts of P04 and P06, in which non-participating fathers and counter-therapeutic grandmothers undermined treatment consistency. Psychoeducation directed at the full family system—not only the primary caregiver—is warranted. This should include structured sessions with fathers, extended family members, and other significant caregivers, addressing the rationale for behavioral strategies and the clinical risks of inconsistent implementation. Research by Moes and Frea (2002) demonstrates that family systems interventions improve behavioral outcomes.

Clinical Recommendation 3 (Enhanced Communication Protocols) is motivated by Section 3.1.3 (Communication Gaps) and the analysis of perceived power asymmetry between caregivers and providers. Recommended practices include brief written home-programming summaries after each session, short in-session caregiver-coaching segments, collaborative goal-setting at scheduled intervals, and structured parent feedback mechanisms that invite rather than merely tolerate caregiver input. These practices support shared decision-making and caregiver self-efficacy.

Clinical Recommendation 4 (Signposting to Economic Supports) is motivated by Section 3.2.2 (Economic and Structural Support), in which participants identified financial assistance as a mechanism for recovering time and enabling more consistent participation. Clinicians should be prepared to connect families with applicable funding mechanisms, including Medicaid waiver programs, state autism funding, employer-based accommodations, and community resources. Providing this information signals that the program recognizes economic barriers and is committed to supporting family participation equitably.

Clinical Recommendation 5 (Embedded Relaxation Routines and Family Therapy Integration) is motivated by caregiver-generated proposals documented in Section 3.2.4, in which P03 recommended pre-session relaxation activities and P04 proposed the integration of family therapy. These recommendations warrant formal evaluation as adjunctive components of ABA service delivery. Ingersoll and Dvortcsak (2010) describe transdiagnostic integration strategies that combine behavioral and family-centered approaches.

### 4.3. Positioning Within Existing Literature

This modest, single-site account contributes contextually to a substantial existing literature on barriers to caregiver engagement in behavioral interventions. The findings align with and extend research on time constraints (Bearss et al., 2015; Karst & Van Hecke, 2012), caregiver stress and burnout (Osborne et al., 2008), and family system influences on treatment engagement (Haine-Schlagel & Walsh, 2015; Brookman-Frazee et al., 2006). What this dataset adds is a direct account of how eight mothers in one South Florida ABA program describe and prioritize these barriers and what they themselves recommend for service improvement.

The contribution is contextual rather than transformative—a detailed, qualitative portrait of engagement within one service setting that may inform practice in similar contexts serving predominantly Hispanic, socioeconomically diverse populations. The theoretical framework of family systems analysis provides a lens for understanding how barriers operate not as individual deficits but as misalignments between service design and family structural realities. This perspective aligns with calls in the literature for family-centered, equity-aware approaches to ABA service delivery.

## 5. Limitations

This study has several substantial limitations that must be considered when interpreting the findings. First, the sample size (*N* = 8) and composition (mothers only, from one service provider, recruited through convenience sampling) substantially limit generalizability. Single-site convenience samples are inherently biased toward those with greater accessibility and engagement, potentially excluding the most disengaged caregivers whose experiences might differ substantially. The perspectives represented here are those of mothers who were willing and able to participate in a research study, which may exclude families facing the most severe structural barriers.

Second, this is a secondary analysis constrained by the data available from the original dissertation study. The original interview protocol was not specifically designed to collect systematic information about child-level variables (age, ASD severity or support needs, language and cognitive level, and behavioral profile) or treatment-level variables (treatment intensity, duration of ABA services, treatment setting, type of caregiver training received, and specific expectations placed on parents by the service model). These variables are not peripheral details; they are likely to strongly shape caregiver participation, perceived burden, communication with providers, and treatment implementation at home. Their absence is a significant limitation that constrains the depth of analysis and our ability to articulate how barriers operate differentially across children with varying support needs or families receiving different ABA intensities.

Third, all participants were mothers. The perspectives of fathers, grandparents, and other caregivers are entirely absent. This limits understanding of how parental involvement varies by caregiver role and family structure. Given that the absence of father involvement emerged as a barrier theme, paternal perspectives on engagement would be particularly valuable.

Fourth, the data are self-reported and retrospective. Participants’ responses may reflect personal perceptions rather than objective measures of involvement, and recall bias may influence accounts of barriers and facilitators.

Fifth, all participants were from one ABA provider in South Florida. Service philosophy, treatment intensity, family education approaches, organizational support, and demographic composition vary widely across ABA programs. Findings may not transfer to programs with different models, intensity levels, geographic locations, or populations.

Finally, while we employed member checking and reflexive bracketing to strengthen credibility, the epistemological limitations of the secondary analysis remain. Conclusions about how barriers operate universally in ABA or how recommendations would generalize to other contexts cannot be drawn. Future research should systematically collect child- and treatment-level data, include diverse caregiver types across multiple service providers, and employ mixed-method designs to validate findings.

## 6. Conclusions

Among eight mothers of children receiving ABA in South Florida, parental involvement was shaped less by individual willingness than by structural and relational conditions: time, family support, communication, and access to economic resources. Motivation, by contrast, was nearly universal and anchored in observed child progress. These results suggest that services can strengthen engagement by treating it as a matter of system design—through clearer caregiver communication, whole-family education, flexible scheduling, signposting to funding, and evaluation of caregiver-proposed additions such as embedded relaxation routines and family therapy.

The family systems perspective helps explain these findings: parental involvement is not merely an individual responsibility but a function of systemic alignment. When service structures, family relationships, economic conditions, and provider–family communication work together, engagement flourishes. When these systems are misaligned—when a father does not support treatment, when employment demands preclude attendance, when communication flows only from provider to parent—even highly motivated mothers struggle to maintain participation.

While this single-site study cannot generalize broadly, its findings align with existing literature on barriers to service engagement among underserved families and offer specific, actionable recommendations for improving practice in community-based ABA settings serving diverse populations. Future research should include fathers and other caregivers, span multiple providers and geographic regions, and test whether such service-design changes measurably improve engagement, treatment fidelity, and child outcomes. By listening closely to caregivers, ABA services can be reshaped around the realities of family life, to the benefit of children with autism and the families who support them.

## Data Availability

The de-identified interview data supporting the findings are available from the author upon reasonable request, subject to ethical restrictions protecting participant confidentiality.

## Appendix A. Semi-Structured Interview Guide

The following nine-item interview guide was used in all participant interviews:

1. Can you describe your general experience as a parent involved in your child’s ABA therapy?
2. What are the main challenges or barriers you have encountered in participating in your child’s ABA treatment?
3. Are there any factors related to your family situation or your daily life that make it difficult for you to participate fully in treatment?
4. What factors have motivated you to remain involved in your child’s ABA therapy despite these challenges?
5. How do you see your role as a parent in contributing to your child’s progress in ABA?
6. Have you noticed any changes in your child’s behavior or development that you attribute to your participation in treatment?
7. Have there been any communication issues between you and your child’s therapists, school, or other service providers? If so, how have these affected your participation?
8. What support—financial, practical, or from your family—would help you participate more fully in your child’s treatment?
9. If you could recommend any changes to the ABA services your child receives, what would those be?

Interviews lasted approximately 30–60 min and were audio-recorded and transcribed verbatim. Prompts were used to encourage elaboration where responses were brief or unclear (e.g., ‘Can you tell me more about that?’ or ‘How did that make you feel?’).
